# Supplementary material for: Prevalence and factors associated with inappropriate anti- diabetic medication therapy among type 2 diabetes mellitus patients at the medical and surgical wards of Mbarara Regional Referral Hospital, Uganda
Source: PLoS One. 2022 Jun 29;17(6):e0270108. doi: 10.1371/journal.pone.0270108 (PMC9242490; doi:10.1371/journal.pone.0270108)
Supplement: Attachment — Submitted filename: Response to Reviewers.docx [file pone.0270108.s001.docx]

Date- 20/05/2022

**Academic Editor, PLOS ONE**

Dear Sir/Madam,

RE: Response to Academic Editor and Reviewers Feedback on “**PVALENCE AND FACTORS ASSOCIATED WITH INAPPROPRIATE ANTIDIABETIC MEDICATION THERAPY AMONG TYPE 2 DIABETES MELLITUS PATIENTS AT THE MEDICAL AND SURGICAL WARDS OF MBARARA REGIONAL REFERRAL HOSPITAL, UGANDA**”

I have received a letter with the comments and suggestions on my manuscript from your committee on 1^st^ May, 2022. I have addressed all the comments in the manuscript and also presented my responses below.

- Responses to Specific comments

1. I revised my manuscript according to PLOS ONE ‘s style requirements
2. I addressed the concerns raised on ethics statement according to the recommendations provided
3. As recommended I removed any funding-related texts from the manuscript and I would like to keep my funding information as it currently appears in the Funding Statement section of the online submission form, which reads as “This work was funded by Pharm-Bio Technology and Traditional Medicine Center (PHARMBIOTRAC).
   The funders had no role in study design, data collection and analysis, decision to publish, or preparation of the manuscript”.
4. I generated ORCID id as recommended
5. I addressed all the comments raised on the figures.
6. I referred to table 4 in my text as recommended and there is no table 5.
7. I improved on my conclusion and discussed on background of diabetes as recommended by reviewer #1.
8. I included the missing abbreviations, missing figures and future prospective of the study.
9. I also worked on formatting errors and grammatical errors.
10. I also tried to improve on result and discussion section of my manuscript.

Sincerely,

Konjit Abebe Nigussie
